# The Western Australian medical schools mindfulness project: a randomised controlled trial

**DOI:** 10.1186/s12909-024-06128-0

**Published:** 2024-10-22

**Authors:** S. Moore, N. Mavaddat, K. Auret, C. Hassed, R. Chambers, C. Sinclair, H. Wilcox, H. Ngo

**Affiliations:** 1 Rural Clinical School of WA, University of Western Australia, Busselton, Australia; 2 Medical School, University of Western Australia, Perth, Australia; 3 Rural Clinical School of WA, University of Western Australia, Albany, Australia; 4 Monash Centre for Consciousness and Contemplative Studies, Monash University, Melbourne, Australia; 5 School of Psychology, University of New South Wales, Sydney, Australia; 6 Rural Clinical School of WA, University of Western Australia, Perth, Australia

## Abstract

**Background:**

Evidence for the longer-term benefits of online mindfulness training for medical students, including in the reduction of stress and improved wellbeing, is limited. This study aimed to evaluate the impact of a novel online mindfulness training program on trait mindfulness, wellbeing and study engagement of medical students at program completion and 6-month follow-up.

**Methods:**

This was a randomised waitlist control study of an 8-week, online, mindfulness-based intervention versus normal curriculum alone for medical students (*N* = 114). The primary outcome measures were the changes from baseline to program completion at Week 8 for self-reported trait mindfulness (Freiburg Mindfulness Inventory), perceived stress (Perceived Stress Scale), self-compassion (Self-Compassion Scale) and study engagement scores (Utrecht Work Engagement Scale for Students). The secondary outcome measures were these score changes from baseline to 6-month follow up. Intervention and control students completed surveys at all three time points. Program adherence (Mindfulness Adherence Questionnaire) was also measured in the intervention group.

**Results:**

The intervention group experienced modest but statistically significant improvements in mindfulness (9%, *p* = 0.0002), self-compassion (5%, *p* = 0.026), and study engagement (4%, *p* = 0.035) from baseline to Week 8. They also reported a sustained improvement of 5% (*p* = 0.017) in mindfulness scores at 6 months. The control group reported no significant changes at Week 8 or 6 Months. Between-group comparisons showed that compared to the control group, the intervention group improved significantly more in mindfulness (*p* = 0.0076), and statistically marginally more in study engagement (*p* = 0.0534) at Week 8. No statistically significant between-group differences were observed at 6 months.

**Conclusions:**

These results add to the small but growing body of evidence suggesting that online mindfulness-based interventions with minimal contact can improve, albeit in modest magnitude, mindfulness and possibly study engagement in medical students for the duration of a mindfulness program. Further refinements to the program may be important to maintain improvements in the longer-term.

**Trial registration:**

Registration number ACTRN12624000783527.

## Introduction

It is well established that medical students experience high levels of stress and burnout [2, 18, 20]. Factors contributing to these high rates of stress and burnout include frequent exposure to patient and family suffering, demanding academic assessments, lack of personal time, competition among peers and poor learning environments [15]. This has consequences such as mental illness and suicide [17, 45, 52, 56] as well as impaired academic performance, cynicism about their profession and academic dishonesty [16]. Further, burnout commonly occurs during junior doctor training, leading to increased medical errors and a reduction in the quality of patient care [44].

One approach to mitigating the problem of medical student stress, burnout and mental illness has been teaching mindfulness to medical students during medical school. Mindfulness is “paying attention, on purpose, in the present moment, non-judgementally” [32, 33]. Mindfulness can be practised formally, i.e., sitting meditation, or informally, i.e., paying attention to everyday tasks and activities. There is a growing evidence base demonstrating that teaching mindfulness to medical students moderates stress levels and improves wellbeing [12, 27]. Hathaisaard et al. [27] conducted a meta-analysis of six high-quality randomised controlled studies comparing the efficacy of mindfulness-based interventions (MBIs) to typical medical curriculum that does not include mindfulness training (*N* = 689) and found a significant improvement in stress measurement scores immediately following the MBIs and at 6-month follow-up in the intervention participants compared to controls. da Silva et al. [12] also conducted a meta-analysis of eight RCTs, of which five were also included in the meta-analysis by Hathaisaard et al. [27], comparing the effectiveness of Mindfulness Based Stress Reduction (MBSR) programs to the usual medical curriculum, and found a significant improvement in mindfulness, psychological wellbeing and stress outcome measures on completion in MBSR participants compared to controls. Traditionally, MBSR programs are 8 weeks long, with 2–3 h of face-to-face instruction in a group, a 7 h intensive and 45 min of home mindfulness practice each day [32, 32]. Cultivating mindfulness is emerging as an important strategy for sustaining medical student wellbeing during the high-pressure journey of medical school training [12, 19, 27]. Notably, only two of the studies [62, 63] included in these meta-analyses involved self-guided, online program delivery, highlighting the need for more research of online mindfulness training programs [12, 19, 27].

There is also literature to suggest that training in mindfulness can lead to increased levels of self-compassion [7, 8, 49]. Self-compassion can reduce stress and optimise coping strategies [1, 49], improve mood [37] and lead to better performance following failure on an academic test [9] all of which can improve medical student wellbeing. Further, research shows that mindfulness can enhance work and study engagement [3, 41] including in medical students [4, 34]. Study or work engagement is generally considered to be inversely correlated with burnout [21]; hence optimising study engagement in medical students may in fact reduce burnout and improve student wellbeing, and vice versa.

Until recently, most mindfulness training in medical schools has been delivered face-to-face by a facilitator via interactive lectures coupled with home practice [31, 34, 42, 43, 60]*.* However, as a result of the recent COVID-19 pandemic, many universities have moved to developing and implementing online teaching methods to deliver their programs [14, 64]. A qualitative study by Dederichs et al. [14] investigating medical students’ perspectives on stress-reduction interventions reported that students found online presentations to be an efficient mode of delivery to reduce stress that allowed balance with study, family and work commitments. Dederichs et al. [14] also found that brief, ongoing, voluntary interventions that are accessible online are perceived by students as preferable to a face-to-face format. Further, the authors suggested that self-management interventions that add significant time to existing classes and workload may in fact increase stress rather than reduce it, suggesting that brevity should be a priority for such interventions.

Evidence for technology-led delivery of mental health interventions is growing. A systematic review of published web-based tools and mobile applications designed for mitigating stress, burnout and suicidality tested in healthcare students and professionals was conducted by Pospos et al. [51]. They identified 14 web-based tools and 22 mobile applications and reviewed their content, format, duration, advantages, and disadvantages. While ten of these tools demonstrated evidence of efficacy, none of the programs were purposely designed for use by healthcare students or professionals. Consequently, Pospos et al. [51] recommended that future research should assess the effectiveness of digital programs specifically among healthcare workers to establish any reductions in stress, burnout and suicidality resulting from the intervention and to evaluate their use to guide improvements in outcomes. Only the web-based cognitive behavioural therapy (CBT) program MoodGYM was found to be evidence-based [51]. Guille et al. [22] found that medical interns who utilised this program experienced a statistically significant reduction in suicidal ideation.

Since the Pospos et al. [51] review, a number of online mindfulness-based interventions (MBIs) have been studied amongst university students [13, 38, 54, 64]. These programs vary from six to eight weeks in duration and related studies have typically measured changes in depression, anxiety, mindfulness, self-compassion and burnout. Findings from these studies indicate that participants of online MBIs who complete most sessions do experience improvements in these measures immediately post-intervention [13, 38, 54, 64]. However, longer term follow-up evaluation beyond a few months has not been conducted in any of these studies, highlighting a gap in the literature.

In addition, further studies have confirmed that MBIs delivered via mobile applications are associated with positive effects on stress and mindfulness levels in university students [30, 50] and employees [53]. One study among registered nurses found that their benefits are comparable to face-to-face MBIs [47], and another that virtual teaching of mindfulness-based skills is as effective as in-person teaching for promoting wellbeing in physician-assistant students [29]. Recent meta-analyses of RCTs evaluating the efficacy of mental-health and mindfulness smartphone apps compared to control conditions in the general population concluded that these apps have a small but significant effect on the symptoms of depression and anxiety and recommend future research to explore the strength and limitations of this literature [23, 40]. Affordability, flexibility and adaptability to the users’ needs have been suggested as underlying reasons for these positive findings for online MBI programs, however further research is indicated to understand how to optimise the efficacy of MBIs for use as a mobile application platform [30], particularly given the growing preference for online resources in the post-COVID era. Further, online mindfulness programs potentially improve access for medical students studying in rural and resource-limited areas. However, it is important to note that on review of real-world objective data on user engagement with mental health apps, only a small percentage of users continue to use these apps for an extended period of time [6], highlighting the need to investigate these limitations further.

Although some studies of mindfulness programs have shown that formal mindfulness practices (e.g., meditation) contribute to more positive health and wellbeing outcomes [10, 28], integration of informal mindfulness practice throughout one’s day has also been demonstrated to be associated with improved health and wellbeing [36, 58]. However, few studies have required participants to formally record both formal and informal (i.e. incorporated into daily activities) mindfulness practice.

In 2016, a pilot project was conducted in our institution to test the feasibility and potential effectiveness of a novel online mindfulness-based training program designed specifically for medical students, with a focus on rurally-located medical students [46]. This pilot demonstrated that an online format was a feasible and low-cost way of delivering mindfulness training to these students. Medical students in the program also showed reduced perceived stress levels and increased self-compassion at 4-month follow up relative to baseline scores [46]. However, this pilot study did not have a control group of students and did not include trait mindfulness and study engagement as additional measures of effectiveness.

Building on our prior work, the aim of the current study was to conduct a randomised controlled trial (RCT) to determine the efficacy of an online MBI for medical students, the “Mindfulness Training Program” (MTP), relative to a waitlist control. Specifically, we measured changes in trait mindfulness, stress, self-compassion and study engagement in a different cohort of medical students at program completion at 8 weeks, and then at 6-month follow-up. Further, measurements of formal and informal mindfulness practice were included to assess adherence to the intervention.

## Methods

### Trial design

**Fig. 1:**
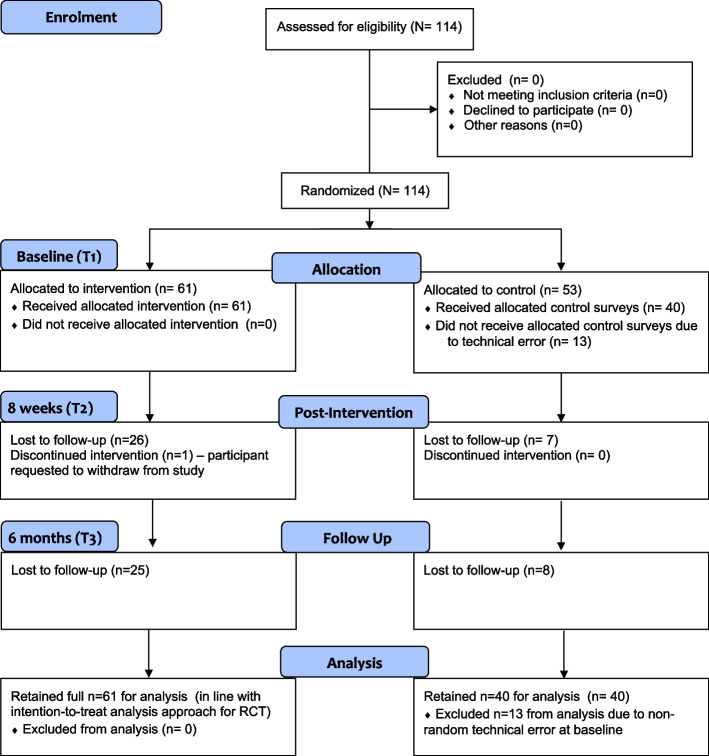
CONSORT 2010 flow diagram

This was a randomised waitlist control study of the MTP. See the Consort Flow Diagram (Fig. 1) for an overview of the study design.

### Participants

Students from all academic years from two Australian medical schools, University of Western Australia (UWA) and University of Notre Dame Fremantle (UNDF), were recruited during a 2-month period (February to March) in 2018, 2019 and 2020. Both universities offer a Doctor of Medicine program to graduate students, with UWA being a public university, and UNDF a private Roman Catholic university. Both pre-clinical (no patient contact) and clinical phase (patient contact) students were invited to participate, including students attending the Rural Clinical School of Western Australia, which selects penultimate year students from UWA, UNDA and, more recently, Curtin University, to complete one year in a rural location. All students were sent an email with an invitation to participate in the study by their medical schools on behalf of the study team. Participation was voluntary and required signed informed consent. Students were informed that the decision of whether to participate would have no impact on their academic marks and that they could withdraw from the study at any time.

### Instruments

*The Kessler Psychological Distress Scale* (K-10; [35] was used to measure psychological distress at baseline. This scale was administered as part of the intake survey to screen for participants at high risk of psychological distress. The K-10 is a widely used, validated and reliable tool used for measuring the severity of symptoms of anxiety and depression an individual has been experiencing in the previous four weeks. Participants respond to each item using a 5-point scale ranging from 1 (none of the time) to 5 (all the time). Scores range from 10 (minimum) to 50 (maximum). Higher scores indicate a higher likelihood of anxiety or depression [35]. Participants who recorded a score of greater than 30 (highest score 36 in this sample) were advised that their score was in the very high range, their potential psychological distress acknowledged and were provided with contact details for local mental health support hotlines as per the research team’s duty of care.

*Freiburg Mindfulness Inventory* (FMI; [61], is a validated and reliable 14-item instrument to measure trait mindfulness. Responses are given on a scale ranging from 1 (rarely) to 4 (almost always). Total possible scores range from 14 to 56; higher scores indicate higher levels of mindfulness [61].

The Perceived Stress Scale (PSS; [11] was used to measure perceived stress. The PSS is a widely used, validated and reliable 10-item instrument, which measures the degree to which situations in an individual’s life are perceived as stressful. Participants respond to each item using a scale ranging from 0 (never) to 4 (very often). Total possible scores range from 0 (minimum) to 40 (maximum); higher scores indicate higher levels of perceived stress [11].

*The Self-Compassion Scale* (SCS; [48] was used to measure self-compassion. The SCS is a validated and reliable 26-item instrument that assesses components of self-compassion. Responses are given on a scale ranging from 1 (almost never) to 5 (almost always). Total possible scores range from 26 to 130; higher scores indicate higher levels of self-compassion [48].

*The Utrecht Work Engagement Scale for Students* (UWES-S; [57] was used to measure study engagement. The UWES-S is a validated and reliable 17-item instrument to assess student engagement with their studies. Responses are given on a scale ranging from 0 (never) to 6 (always/every day). Total possible scores range from 0 to 102; higher scores indicate higher levels of study engagement [57].

*The Mindfulness Adherence Questionnaire* (MAQ; [26] is a newly developed, validated and reliable 12-item self-report of adherence to mindfulness-based practice within the past week. The first two items measure quantity of formal practice in terms of frequency and average duration (in minutes). The final ten items measure the quality of mindfulness practice, both formal and informal. Practice quality is the level to which a person is practising mindful attention and attitude during meditation and informal practice. Items are scored on a 7-point Likert-scale ranging from 0 (never) to 6 (always). Total possible scores for the quality of formal and informal practice range from 0 to 24 and 0 to 36 respectively. Higher scores reflect higher practice quality [26].

### Interventions

The MTP was delivered over eight weeks between March and May in 2018, 2019 and 2020. It included eight mini-lectures (10 min duration each) focusing on various applications of mindfulness relevant to medical students and eight guided mindfulness meditation sessions (5 min duration). Specific content of the mini-lectures and mindfulness practices is shown in Table 1. One mini-lecture was emailed as an audiovisual recording to participants at the beginning of each week and one mindfulness meditation was sent via SMS as an audio recording daily from Monday to Friday. On completion of the 8-week program, participants had ongoing access to all the mini-lectures and mindfulness recordings and were encouraged to utilize these resources over the following 6-month period. The control group received the usual curriculum, which contained no formal mindfulness training, for the duration of the study and were offered the opportunity to participate in the mindfulness program on completion of their participation in the trial as an incentive to participate in the study.

**Table 1:** Content of MTP

**Mini-lectures**	**Mindfulness practices**
Week 1. Multi-tasking vs effective task switching	Week 1. Body scan
Week 2. Stress reduction and performance	Week 2. Mindful breathing
Week 3. Reducing distraction and procrastination	Week 3. Thought-labelling
Week 4. Mindful communication	Week 4. Mindful listening
Week 5. Regulating emotions	Week 5. Working mindfully with emotions
Week 6. Compassion	Week 6. Loving kindness
Week 7. Self-compassion	Week 7. Self-compassion
Week 8. Mindful use of technology	Week 8. Mountain meditation

### Outcomes

#### Primary outcome measures

The primary outcome measures were changes in trait mindfulness (FMI), perceived stress (PSS), self-compassion (SCS) and study engagement (UWES-S) survey scores from baseline (T1) to program completion (T2). These survey measures were selected as they have been repeatedly assessed as expected outcomes of mindfulness training; further, there is no consistent evidence to consider one measure more important than another in evaluating outcomes of mindfulness interventions.

#### Secondary outcome measures

The secondary outcome measures were the changes in trait mindfulness (FMI), perceived stress (PSS), self-compassion (SCS) and study engagement (UWES-S) survey scores from baseline (T1) to 6-month follow-up (T3).

#### Other data

Other data collected at baseline via email survey included demographics (age, gender, ethnicity, home university and year of medical school), details of any prior training in mindfulness, whether participants already had a regular mindfulness practice and a measure of their current psychological distress via the K-10 survey [35]. Among the intervention participants, measures related to mindfulness practice including quantity of formal practice and quality of formal, informal, and overall practice, were also assessed to report on adherence to the intervention.

During the intervention, the MAQ [26] was administered weekly to intervention participants via email to record the frequency, duration, and quality of formal and informal mindfulness practice during the previous week. At the completion of the MTP, control participants were asked to report the frequency and duration of any self-directed formal mindfulness practice they had undertaken during the previous 8 weeks. At the 6-month follow-up (T3), all participants were asked to report on the frequency and duration of any mindfulness practice they had undertaken between completion of the MTP (T2) and the 6-month follow-up (T3).

### Sample size

Sample size calculations were performed using the PROC POWER procedure in SAS for each of the primary outcome measures, namely the score changes in trait mindfulness, stress, self-compassion, and study engagement, from baseline to program completion (T1 to T2). Assuming negligible (i.e., near zero) changes within the control group, and modest improvements, of approximately 10% of the baseline score, within the intervention group, a minimum of *N* = 60 and *N* = 40 would be required for the Intervention group and the Control group, respectively. All minimum planned sample sizes were expected to afford a statistical power of at least 0.8 and statistical significance level of 0.05.

### Randomisation and blinding

Randomisation codes were generated using the PROC PLAN procedure in SAS. Randomisation was run after each yearly (2018–2020) recruitment was completed. Randomisation was run separately for male and female participants, as a ‘surrogate’ for stratification, due to (a) previous evidence for differential effects of mindfulness training in each gender [55], and (b) disproportionately more female participants in our sample. Blinding of participants to the investigator (SM) and the statistician (HN) was not feasible in this RCT due to the weekly nature of the intervention provision and weekly follow-up via the MAQ for the Intervention group over the course of eight weeks, versus follow-up at only T1, T2 and T3 for the control group.

### Statistical methods

Data on outcome measures were first summarised descriptively. The statistical significances of before-after within-group score changes on trait mindfulness, perceived stress, self-compassion and study engagement were assessed using paired t-test. Measures of mindfulness practice for intervention participants were also described using mean and standard deviations. Statistical predictors of primary outcomes, namely score changes from T1 to T2, were assessed using generalized linear modelling (GLM). Candidate statistical predictors considered in the GLM models included the participants’ randomized group, age, gender, ethnicity, year of study, prior exposure to mindfulness, and current practice of mindfulness.

Study data were collected and managed using the REDCap electronic data capture application hosted at University of WA [24, 25]. Data were then exported to Excel for cleaning and then SAS (version 9.4) for statistical analyses. Consistent with the randomised controlled design, intention-to-treat (ITT) analysis approach was followed. To gain a deeper insight into the potential impact of missing data due to loss-to-follow-up (LTFU), a sensitivity analysis was also conducted for the four primary outcome measures, comparing results from the ITT analytical approach to the per-protocol (PP) approach. Statistical significance was set at α = 0.05.

## Results

### Participant flow

An overview of participant flow through the study is provided in Fig. 1. A total of 114 medical students between years 1 – 4 of their medical degree were recruited into the study and completed baseline measures. Participants who did not return follow-up data at program completion and/or 6 month follow up were considered lost to follow up, although their data were still included in analyses, as per the ITT approach, with missing data at follow-up imputed to denote ‘zero’ change, compared to baseline. Of the 53 control arm participants, all 13 from the 2018 cohort did not return any follow up survey data as they did not receive the email link due to an error in the REDCap survey setup; therefore, data on these 13 participants were excluded from all analyses. One intervention participant requested to withdraw from the study mid-program. Their data was retained up until their withdrawal date.

### Baseline data

As shown in Table 2, the two randomised groups were relatively comparable across most key characteristics at baseline. Female participants made up three quarters of the study population; and students located in a rural location for their academic year comprised between 25–34% of the cohort. Most participants were of Caucasian ethnicity (75%), with Asian students comprising 15% of the cohort. Students from all four years of the medical degree were relatively equally represented in the intervention and control groups. Just over one third of students reported having had some prior exposure to mindfulness practice before commencing the program, including the Monash Health Enhancement Program delivered to Notre Dame University medical students, the Smiling Mind app, and yoga.

**Table 2:** Description of two randomised groups at baseline

**Characteristics**		**Intervention (*n*=61)**		**Control (*n*=40)**		*p-value*
		*Mean*	*SD*	*Mean*	*SD*	
***Age (in years)***^a^		*25.0*	*4.9*	*25.5*	*7.9*	*0.7483*
***K10 score***^a^		*19.2*	*5.3*	*19.6*	*6.2*	*0.7648*
***PSS score***^a^		*14.8*	*6.0*	*15.6*	*7.2*	*0.5639*
***SCS score***^a^		*83.5*	*18.8*	*84.3*	*19.3*	*0.8282*
***FMI score***^a^		*33.3*	*7.7*	*35.0*	*8.8*	*0.3110*
***UWES score***^a^		*59.6*	*14.6*	*66.9*	*15.6*	*0.0176*
		Frequency	%	Frequency	%	*p*-value
**Gender**	Male	16	26%	10	25%	0.8901
	Female	45	74%	30	75%	
**Ethnicity**	Asian	9	15%	6	15%	0.4319
	Caucasian	44	72%	32	80%	
	Other	8	13%	^b^		
**RCS**	Yes	21	34%	10	25%	0.3169
	No	40	66%	30	75%	
**Year of Study**	1	13	21%	16	40%	0.2547
	2	16	26%	8	20%	
	3	23	38%	11	28%	
	4	9	15%	5	13%	
**University**	Uni1	38	62%	20	50%	0.4736
	Uni2	22	36%	19	48%	
	Uni3	^b^		^b^		
**Prior mindfulness**	Yes	23	38%	15	38%	0.9834
	No	38	62%	25	63%	

*K-10* Kessler Psychological Distress Scale (10-item version), *PSS* Perceived Stress Scale, *SCS* Self Compassion Scale, *FMI* Freiburg Mindfulness Inventory, *UWES* Utrecht Work Engagement Scale, *RCS* Rural Clinical School

^a^Continuous variables, in italics, are presented in mean & standard deviation (SD), with between-group comparisons conducted using independent two sample t test

Other (categorical) variables are presented in frequency and percentage, with between-group comparisons conducted using logistic regression

^b^Small counts of less than 6 have not been reported

At T1, most participants across the two groups reported a moderate level of psychological distress according to their mean K10 scores (mean = 19.2 points) [35]. Scores of 10–20 are considered moderate on the K10 survey [35].

Table 2 further shows the two groups returned comparable scores for their FMI, PSS and SCS scores at T1. The only statistically significant difference at T1 was that control participants on average scored higher than their intervention counterparts on the UWES (i.e., reported higher levels of study engagement) by approximately 7 points (*p* = 0.0176), or a 12% difference. Internal consistency reliability, measured via Cronbach’s alpha, was in the high range for each of the five scales used at baseline: 0.87 for K10, 0.91 for FMI, 0.88 for PSS, 0.95 for SCS, and 0.93 for UWES.

### Primary Outcomes: Score changes on Freiburg Mindfulness Inventory (FMI) Perceived Stress Scale (PSS), Self-Compassion Scale (SCS) and Utrecht Work Engagement Scale (UWES) from Baseline (T1) to Program Completion (T2)

Table 3 below shows that intervention participants’ mean FMI scores improved significantly at T2 by 2.9 points (approximately 9% increase from baseline, *p* = 0.002), as did mean SCS scores by 4.4 points (5%, *p* = 0.026) and mean UWES scores by 2.3 points (4%, *p* = 0.035). Intervention participants’ PSS scores did not change significantly at T2 compared to T1. There were no significant changes within control group participants in any of the outcome scores at T2. Between-group comparisons (Table 4) show significantly greater improvement at T2 in FMI scores (mean difference 2.8, CI 0.8, 4.8; *p* = 0.0076) and some weak (i.e., statistically marginal) evidence for greater improvement in UWES scores (mean difference 3.3, CI 0.04, 6.6; *p* = 0.0534) in the Intervention group, compared to the Control group. Between-group differences in score changes at T2 were not significant for PSS (*p* = 0.1165) and SCS (*p* = 0.1349).

**Table 3:** Scores on Perceived Stress Scale (PSS), Self-Compassion Scale (SCS), Freiburg Mindfulness Inventory (FMI), and Utrecht Work Engagement Scale (UWES), at Baseline (T1), Week 8 (T2), and 6-month (T3) follow-up

**Group**	**Scale**	**T1**		**T2**		**T3**		**Within-group difference: T2-T1**^‡^		**Within-group difference: T3-T1**^‡^	
		mean	SD	mean	SD	mean	SD	estimate (CI)	*p*-value	estimate (CI)	*p*-value
Intervention	PSS	14.8	6.0	14.5	6.1	15.3	5.7	0.4 (-0.6, 1.4)	0.451	-0.4 (-1.5, 0.7)	0.441
(n_I_ = 61)	SCS	83.5	18.8	87.9	18.4	85.4	17.6	4.4 (0.6, 8.3)	0.026*	2.0 (-0.7, 4.7)	0.151
	FMI	33.3	7.7	36.2	8.0	35.0	7.9	2.9 (1.5, 4.4)	0.0002**	1.8 (0.3, 3.2)	0.017*
	UWES	59.6	14.6	61.9	14.1	60.6	14.2	2.3 (0.2, 4.4)	0.035*	1.1 (-1.2, 3.3)	0.354
Control	PSS	15.6	7.2	16.6	6.9	16.6	6.8	-1.0 (-2.4, 0.5)	0.186	-1 (-2.6, 0.6)	0.224
(n_C_ = 40)	SCS	84.3	19.3	84.6	19.8	84.2	19.5	0.3 (-3.2, 3.7)	0.872	-0.1 (-3.7, 3.5)	0.955
	FMI	35.0	8.8	35.1	7.8	35.9	8.4	0.2 (-1.0, 1.4)	0.801	0.9 (-0.4, 2.1)	0.172
	UWES	66.9	15.6	66.0	15.5	66.2	16.7	-1.0 (-3.6, 1.6)	0.449	-0.7 (-4.1, 2.7)	0.682

*Estimate *Estimate of within-group Mean Difference, *CI* 95% Confidence Interval of Estimate

^‡^The three scales SCS, FMI and UWES measure "positive" constructs, therefore a "desired" change would be an increase in score from T1 to T2 or to T3. By contrast, the PSS scale measures a "negative" construct; therefore a "desired" change would be a decrease in score from T1 to T2 or to T3 within-group score differences, or score changes, for all the four outcome scales have been calculated such that numerically positive differences reflect 'desired' changes

^*^indicates effect significant with *p* < 0.05

**indicates effect significant with *p* < 0.001

All statistics are calculated using the intention-to-treat approach

**Table 4:**
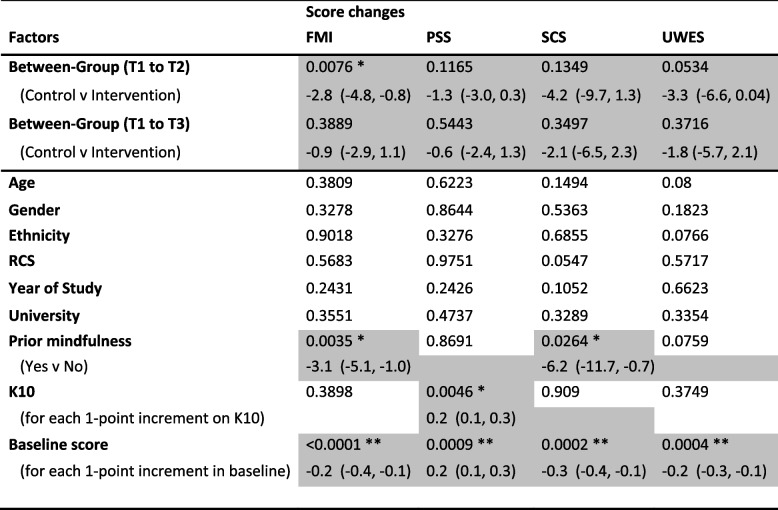
Statistical significance of between-group difference in score changes (from Baseline (T1) to Week 8 (T2) or 6 month (T3)), and other factors

*K-10* Kessler Psychological Distress Scale (10-item version), *PSS* Perceived Stress Scale, *SCS* Self Compassion Scale, *FMI* Freiburg Mindfulness Inventory, *UWES* Utrecht Work Engagement Scale

The first block presents the results for the Randomized Group factor (ie between-group comparison), which forms the study’s primary and secondary interest. The second block presents the results for other factors

Statistics presented in this table are mostly p-values for the respective factors, derived from Generalized Linear Modelling (GLM) approach

Cells shaded in grey contain further information, including the mean difference estimate and associated 95% Confidence Interval. These further details are shown for: (a) the primary outcomes (i.e., between-group difference in score changes from T1 to T2) and secondary outcomes (i.e., between-group difference in score changes from T1 to T3), regardless of statistical significance; and (b) other factors that reach statistical significance (i.e., *p*<0.05 *, or *p*<0.001 **)

Results from the sensitivity analysis, comparing the ITT versus PP analytical approaches, are shown in Table 5. The two approaches returned similar results for the FMI and PSS outcomes; whereas the results for SCS and UWES were slightly more favourable for the Intervention group, with the PP approach (compared to the ITT approach).

**Table 5:** Summary descriptive statistics for Mindfulness Adherence Questionnaire (MAQ) across the 8-week program for the intervention group (*N* = 61)

**Variable**	**Minimum**	**Median**	**Maximum**	**Mean**	**Std dev**	**Std error**
**Average Quantitative (in minutes)**	0	12.5	37.6	13.5	10.5	1.3
**Average Qualitative**	0	23.4	48.8	23.0	14.3	1.8
**Average Qualitative: Formal**	0	9.8	20.5	9.6	5.8	0.7
**Average Qualitative: Informal**	0	13.8	28.3	13.4	8.6	1.1

### Secondary Outcomes: Score changes on Freiburg Mindfulness Inventory (FMI), Perceived Stress Scale (PSS), Self-Compassion Scale (SCS) and Utrecht Work Engagement Scale (UWES) from Baseline (T1) to 6-month follow-up (T3)

Within the Intervention group, improvements in mean FMI score were maintained at T3, up by 1.8 points (*p* = 0.017) from T1, but not for SCS (*p* = 0.151) nor UWES (*p* = 0.354) scores. There were no significant changes within control group participants in any of the outcome scores between T1 and T3. Between-group comparisons showed no statistically significant difference for any of the four secondary outcomes (Table 4).

The remaining part of Table 4 presents the profile of the primary outcomes – namely score changes on FMI, PSS, SCS, and UWES at T2 – by potential predictors. For FMI and SCS, improvements were lesser among those with prior exposure to mindfulness training or practice, and among those with higher baseline scores on FMI or SCS, respectively. For PSS, those with higher psychological distress (K10) or higher perceived stress (PSS) at baseline improved more at T2, compared to peers who reported less distress or stress at baseline. The baseline effect was also present for UWES score changes at T2.

### Adherence to mindfulness practice and its correlations with primary and secondary outcome measures

Table 5 below shows a descriptive summary of the MAQ scores across the 8-week program for the intervention group participants. Mean total duration of formal practice per week over the course of the 8-week program was 13.5 min (SD 10.5). For each of the measures of quality of formal, informal and combined (formal and informal) mindfulness practice, the results were highest in weeks 1 and 2 of the program and trended down to the lowest in weeks 5 to 7 of the program.

Higher quality in mindfulness practice was a predictor of improvement in self compassion scores at program completion, while higher quantity and quality in practice adherence were predictive of improvement in trait mindfulness, and higher quality in informal mindfulness practice predicted improvement in study engagement. At 6-month follow-up, higher quality, especially of informal practice, was a predictor of sustained improvement in trait mindfulness. Extra practice between program completion and 6-month follow-up was a predictor of improvement of trait mindfulness (FMI) at 6-month follow-up.

## Discussion

The main objective of this randomised control study was to evaluate the short- and longer-term impacts of a novel, minimal contact, online mindfulness training program on levels of trait mindfulness, perceived stress, self-compassion and study engagement among participating medical students compared to controls. We found statistically significant improvements in trait mindfulness, self-compassion, and study engagement scores within the intervention group at completion of the 8-week program, while between-group comparisons revealed significantly greater improvement in mindfulness scores and some indication for improvement in study engagement scores for the intervention group participants compared to control participants at completion of the 8-week program. At 6-month follow up, only improvements in trait mindfulness were sustained within the intervention group. No statistically significant results were observed for the control group. Between-group comparisons showed no statistically significant difference for any of the four secondary outcomes at 6-month follow-up.

Our finding that this online MTP is associated with short-term improvement in self-reported trait mindfulness adds to the growing body of evidence that online delivery can be an effective method to train medical students in mindfulness [13, 30, 38, 46, 50, 51, 64]. Although a pilot study of this online MTP in a group of self-selected participants yielded significant improvements in perceived-stress and self-compassion scores at 4-month follow-up, the modest changes in trait mindfulness, self-compassion and study engagement scores detected within the intervention group in this current study suggest that more intensive and/or sustained interventions could be required to yield more significant and longer-term changes in trait mindfulness, self-compassion and study engagement levels. Further, the difference in recruitment approach, ie self-selection vs randomisation, may have played a role.

To our knowledge, our RCT is the first to measure self-compassion score changes in medical students participating in an online MTP. Several factors may help explain why the small but statistically significant changes in this measure occurred at program completion but were not maintained at 6-month follow-up in our study. Firstly, our program included only one short mini-lecture and one week of guided practice focussing on self-compassion in week seven of the program, which may not have been sufficient to lead to significant, longer-term changes in self-compassion scores. There is strong evidence that online programs can increase self-compassion, but these interventions have tended to include much larger amounts of self-compassion-specific content [39]. There is some evidence that programs like MBSR, which are more mindfulness-focused and include relatively small amounts of self-compassion content can increase self-compassion compared to a control group [59], but this program was delivered face-to face rather than online. Future research should vary the amount of self-compassion content and contrast online and face-to-face delivery methods to further elucidate the amount and format of self-compassion training necessary for producing significant increases.

Our finding that intervention participants’ study engagement scores improved significantly at program completion is a potentially encouraging finding for this online mindfulness intervention in a medical student cohort. Study engagement has been found to improve in studies of face-to-face MBIs with medical students [4, 34]. Although the magnitude of change was modest, to our knowledge this is the first study that has been performed assessing the impact of an online program on study engagement that has found some positive change. As noted by [34] improved study engagement may be associated with reductions in burnout, which may provide further benefits for mental health and wellbeing of medical students. The underlying factors contributing to these changes in study engagement could include reduced distraction, anxiety and ruminative cognitive patterns that promote avoidance and procrastination, but further investigation is needed to determine if this is indeed the case. According to post-hoc analysis in other studies of the UWES-S, the Absorption sub-scale of the UWES-S changes little as a result of mindfulness training, thus potentially impacting the overall outcome of the scale [5]. It would be expected that mindfulness should increase vigour and dedication to study, however items on the Absorption subscale such as “I can’t stop thinking about study” are not congruent with mindfulness. Each of these factors may have contributed to the modest improvements in the study engagement scores that were not maintained at 6-month follow-up, despite the potential benefits of mindfulness training to study engagement.

The results from our current study showing that this MTP was not associated with improvements in self-reported perceived stress scores despite improvements in trait mindfulness scores are different to those reported in other RCTs examining online MBIs with medical students, who did detect improvements in self-reported stress scores [54, 62, 63]. Despite these improvements in stress scores, two of these studies found no significant difference in trait mindfulness scores, measured by Five-Facet Mindfulness Questionnaire, between the intervention and control groups at post-intervention follow-up [54, 63]. Explanations for the differences between our findings in this RCT and those of other similar RCTs with regards to perceived stress and mindfulness score changes include the variation in content between the different programs, different platforms over which the online programs were delivered, the duration of the intervention and the timing of the program delivery during the academic year. Yang et al. [63] studied a mobile mindfulness meditation application, Headspace, using a waitlist control over 30 days; Warnecke et al. [62] studied an 8-week intervention utilising custom-designed guided mindfulness practices on audio CD; Ritvo et al. [54] studied an 8-week web-based mindfulness-based intervention. Further, different stressors may have existed for the different cohorts of students that were not measured. For example, there was significant attrition of participants who completed follow-up data in 2018 and 2019, in contrast to minimal attrition in 2020, which coincided with the COVID-19 pandemic. One might speculate that the 2020 cohort maintained engagement with the program due to extreme circumstances of stress and uncertainty.

Further learnings from our study beyond our primary and secondary outcomes include the findings that higher quality mindfulness practice was a predictor of improvements in self-compassion scores and trait mindfulness scores at program completion. High quality informal mindfulness was also predictive of improved study engagement at program completion; this may be taken to suggest that informal mindfulness practice is particularly helpful for enhancing medical students’ engagement with their studies. In addition to this, higher quality informal mindfulness practice was a predictor of sustained improvement in trait mindfulness at 6-month follow-up, which suggests the enduring and long-lasting effect of informal practice. Further, continuing to practice mindfulness between program completion and 6-month follow-up was a predictor of improvement of trait mindfulness at 6-month follow-up, again suggesting the enduring and long-lasting effect of ongoing mindfulness practice. Examining adherence to online MBIs is necessary for understanding the relationship between mindfulness practice and outcome measures, however measurements of adherence in other studies have not been comprehensive.

Previous studies have shown a positive correlation between the adherence to formal and informal mindfulness practice during online MBIs and improved mindfulness scores, self-compassion scores [13, 38] and reduced stress scores [58]. This supports our findings that perceived stress scores correlated positively with quantity of adherence to mindfulness practice. However, unlike these previous studies, our study also used the MAQ to directly measure the *quality* of both formal and informal home practice adherence (i.e., how much participants were actually cultivating mindful qualities such as present-moment focus, acceptance and nonjudgment while engaged in formal and informal practices) and found that the quality of home practice was associated with positive changes in trait mindfulness and self-compassion. This finding suggests that the quality and type of home practice, in particular informal mindfulness practice, has an impact on wellbeing measures. As noted by Shankland et al. [58] there are a number of hypotheses that have been suggested to explain the underlying mechanisms for the efficacy of informal mindfulness, including improved emotional regulation and better balance of mental and sensory information processing in the brain, leading to cognitive and psychological flexibility and adaptation. Further, Danilewitz e al. [13] hypothesised that other features of mindfulness programs, such as group unity and peer encouragement, may be important for optimising student engagement and adherence, particularly for online programs. Future studies could investigate these hypotheses to deepen our understanding of the differential and synergistic effects of formal and informal mindfulness practice as part of online mindfulness training programs.

### Strengths and limitations

This study had three major strengths. First, ours was a randomised controlled trial, which minimises selection bias, allows direct comparison of the intervention to the usual medical school curriculum and minimises confounding factors. Second, we included measures of both formal and informal mindfulness practice using a validated Mindfulness Adherence Questionnaire. Third, our follow up duration of up to 6 months is unique for randomised controlled studies of mindfulness programs for medical students.

There were also limitations to this study. We had a low response rate, with 5% of eligible participants enrolling in the study. The program facilitator was the principal investigator, which may have positively affected participant self-report scores. However, the fact that there was no face-to-face interaction between the facilitator and participants may have reduced this effect. The exclusion of 13 control participants from our data analysis due to technical error may have also impacted our results. As with several other studies in the field before us, we relied on self-report instruments to evaluate the program [54, 62, 63]. We did not gather data regarding whether participants were practicing other formal mindfulness activities unrelated to the MTP during the 8-week program, which may have impacted our results. Future researchers may wish to include objective measures, such as academic grades and attendance at university classes, as proxy measures of mindfulness on study engagement. Engaging local medical educators to assist with participant recruitment may also help boost response rates.

### Implications for future practice and research

This study suggests that self-directed, low-cost, minimal contact online mindfulness training programs can be a feasible alternative to face-to-face MBIs for medical students where it is not possible to deliver a face-to-face program. However, the lack of lasting, long-term effects on self-compassion and study engagement and the absence of changes in perceived stress raises questions about how to increase the longevity of these effects.

Our findings that improvements in mindfulness and self-compassion scores were lesser among those with prior exposure to mindfulness training or practice suggests that online MBIs such as ours may be most beneficial to medical students who have no previous experience with mindfulness. Medical schools should consider this when promoting such programs to their students as this may improve participation in non-mandatory programs. Similarly, our findings that medical students with higher psychological distress and perceived stress scores at baseline improved their perceived stress scores by the greatest amount by the end of the 8-week program suggests that medical schools should preferentially encourage medical students who identify as being stressed and in distress to participate in online MBIs.

Future research studies could directly compare face-to-face with online MBIs to validate the effectiveness of online programs such as ours. In addition, comparing the efficacy of different online programs with regards to duration, frequency, content and follow up length, would provide valuable insights into the strengths and weaknesses of various online MBIs. Low-cost strategies for sustaining positive effects in the longer-term could include incorporating peer-to-peer interactions either online or face-to-face and providing ongoing prompts to undertake mindfulness practice via the online platform. Finally, the ease of delivery of this online program to a large cohort of students makes it an attractive way to include mindfulness training as a standardised but not mandatory offering as part of a medical school curriculum.

## Conclusion

This randomised controlled study demonstrated that a brief online mindfulness program for medical students led to statistically significant improvements in trait mindfulness, in the intervention group compared to the control group on completion of the 8-week program group. We demonstrated that participants who were more committed had more benefits, evidenced by the predictive relationship of the quality of mindfulness practice for the outcome measures. Future innovations could focus on how to support adherence in order to sustain longer term improvements in mindfulness and demonstrate impact on perceived stress, self-compassion, and study engagement as well as trial modifications to the program duration and frequency of mindfulness practice.

## Data Availability

The data that support the findings of this study are not openly available due to reasons of sensitivity and are available from the corresponding author upon reasonable request. Data are located in controlled access data storage at the University of Western Australia.
